# Estimation of indices of health service readiness with a principal component analysis of the Tanzania Service Provision Assessment Survey

**DOI:** 10.1186/s12913-015-1203-7

**Published:** 2015-12-03

**Authors:** Elizabeth F. Jackson, Ayesha Siddiqui, Hialy Gutierrez, Almamy Malick Kanté, Judy Austin, James F. Phillips

**Affiliations:** Heilbrunn Department of Population and Family Health, Columbia University Mailman School of Public Health, 60 Haven Ave, Suite B-2, New York, NY 10032 USA; Department of Family Medicine, Ichan School of Medicine at Mount Sinai, New York, NY USA

**Keywords:** Service provision assessment, Readiness, Situation analysis, Health system, Principal component analysis, Service provision assessment survey, Tanzania

## Abstract

**Background:**

Service Provision Assessment (SPA) surveys have been conducted to gauge primary health care and family planning clinical readiness throughout East and South Asia as well as sub-Saharan Africa. Intended to provide useful descriptive information on health system functioning to supplement the Demographic and Health Survey data, each SPA produces a plethora of discrete indicators that are so numerous as to be impossible to analyze in conjunction with population and health survey data or to rate the relative readiness of individual health facilities. Moreover, sequential SPA surveys have yet to be analyzed in ways that provide systematic evidence that service readiness is improving or deteriorating over time.

**Methods:**

This paper presents an illustrative analysis of the 2006 Tanzania SPA with the goal of demonstrating a practical solution to SPA data utilization challenges using a subset of variables selected to represent the six building blocks of health system strength identified by the World Health Organization (WHO) with a focus on system readiness to provide service. Principal Components Analytical (PCA) models extract indices representing common variance of readiness indicators. Possible uses of results include the application of PCA loadings to checklist data, either for the comparison of current circumstances in a locality with a national standard, for the ranking of the relative strength of operation of clinics, or for the estimation of trends in clinic service quality improvement or deterioration over time.

**Results:**

Among hospitals and health centers in Tanzania, indices representing two components explain 32 % of the common variance of 141 SPA indicators. For dispensaries, a single principal component explains 26 % of the common variance of 86 SPA indicators. For hospitals/HCs, the principal component is characterized by preventive measures and indicators of basic primary health care capabilities. For dispensaries, the principal component is characterized by very basic newborn care as well as preparedness for delivery.

**Conclusions:**

PCA of complex facility survey data generates composite scale coefficients that can be used to reduce indicators to indices for application in comparative analyses of clinical readiness, or for multi-level analysis of the impact of clinical capability on health outcomes or on survival.

**Electronic supplementary material:**

The online version of this article (doi:10.1186/s12913-015-1203-7) contains supplementary material, which is available to authorized users.

## Background

Tanzania is on target to achieve the Millennium Development Goal (MDG) 4 of reducing child mortality by two-thirds by the end of this year [1]. Evidence suggests that this trend is due at least in part to successful health system investments. However, despite significant gains in systems development, constrained access to primary health services and quality of care remains as a challenge. According to United Nations estimates, Tanzania ranks among the highest 10 % in the global ranking of maternal mortality, 27th in the world for under-five mortality, and among the lowest in the global ranking of countries by contraceptive prevalence [2–4]. Only one third of the mandated skilled health workforce is actually employed by the Ministry of Health and Social Welfare (MOHSW) owing to training lapses, high turnover, and low levels of compensation. Moreover, a range of challenging ancillary manpower problems compound staff shortages such as inadequate training, and inappropriate deployment leading to underdevelopment of outreach services [5]. Contributing to these challenges are lapses in supporting systems that lead to insufficient equipment, medicines and supplies [6]. The concatenation of these problems is particularly challenging at the lowest tier of health facilities, the dispensary, where the quality of care is so poor that potential clientele often bypass the most convenient facility [7, 8]. Compounding limitations of routine primary health care are weaknesses in the referral, triage, logistics, and care systems that prevent the implementation of adequate emergency health services [5].

In response, the MOHSW has engaged in evidence-based initiatives to strengthen health systems and to test the impact of their strategies on health outcomes. In 2006, the MOHSW and the National Bureau of Statistics conducted the Tanzania Service Provision Assessment (TSPA) Survey [9] to evaluate the capacity of the Tanzanian healthcare system to support services for child health, family planning, maternal health, and specific infectious diseases such as sexually transmitted infections, tuberculosis, malaria, and HIV/AIDS. Using data from a representative sample of 611 health facilities dispersed throughout all 26 regions of the country, the survey included facilities representing all levels of the health system—i.e., hospitals, health centers, and dispensaries. Stand-alone facilities offering HIV/AIDS services and various types of operational schemes, including government-sponsored, private for-profit, parastatal, and faith-based initiatives, were also included.

The TSPA was launched to supplement household-based data from the 2004–2005 Demographic and Health Survey (DHS), which provides routine information on health status and the utilization of health services at the national level. Although the TSPA was intended to provide useful descriptive information on health system functioning to supplement the DHS, SPA data cannot be linked to the DHS because there is no way to determine whether the catchment areas of SPA facilities contain the households of individuals interviewed in the DHS. For this reason, research linking service system information from facilities cannot be used to assess the effect of clinical capability on health or survival.

Data reduction challenges also constrain the utilization of SPA data for health research. As a tool for generating implementation evidence, the TSPA generates several hundred indicators of health systems capability at a given point in time. Without means of reducing these indicators into interpretable indices that can be monitored with time and used to gauge system performance, the TSPA becomes a “one off” snap shot of problems without prospects that its information base can be a tool for health systems strengthening. In all, the TSPA compiled data on over 3200 indicators of service functionality from a 283 page questionnaire addressing themes about staffing patterns and availability, functioning equipment, essential drugs, and other elements of service readiness, assessed in a single episode of data compilation. A voluminous 700 page report presented tabulation of frequency distribution, with many variables classified as having 10 or fewer facilities. Not surprisingly, the deluge of indicators represents a barrier to practical utilization of TSPA results.

This data complexity leads to a further limitation: The absence of tractable indicators of system capacity offsets any prospect for linking TSPA data with survey data or the utilization of TSPA data for health system monitoring or management, preventing possible investigation of the impact of health system readiness on health indicators in catchment area populations or the use of indicators for monitoring trends in facility readiness. Moreover, the absence of TSPA-based indices prevents the utilization of indicators for monitoring health system performance or readiness. Efforts to strengthen health system functioning are therefore pursued without relying on SPA data sets to guide inference about implementation success.

Nonetheless, the TSPA configuration of information and indicators share a common purpose that includes representing elements of the readiness of health facilities to provide essential services. This paper aims to extract indices based on this common variance, not only to facilitate the interpretation of SPA information, but also to set the stage for further analysis that will link indices with healthy facility survey data in the context of a randomized controlled trial of community health worker impact on child mortality in rural Tanzania. This study, titled *Connect* [10], will utilize the measures of service readiness generated in the present research in order to assess health system readiness at the health facilities that serve the *Connect* study population. Each of the 200 SPA variables selected to represent the WHO building blocks were included in health facility surveys of each facility in the *Connect* study area. These variables will be used to create indices of system readiness in the *Connect* study area. Indices will be included in multivariate analyses of *Connect* impact in order to control for the confounding effect of health system readiness on measurement of community health worker efficacy. Thus, our analysis aims to illustrate a method of data utilization that could enhance the value of service provision assessment surveys for policy.

In summary, our paper aims to demonstrate a method that could be used to improve the utilization of data of the type that the TSPA represents. We base our analysis on the proposition that the various indicators collected in the TSPA are intended to represent a health system that is functioning with some measureable degree of capability. We aim to identify this commonality with “principal components analysis,” demonstrate association of indicators in the TSPA with this index, and posit uses for the index that could be applied to the monitoring of health system strength in Tanzania. Indices developed here could be used to control for readiness to provide care at the nearest health facility level, in multilevel hierarchical analyses assessing the public health impact of strategies for improving the quality or intensity of care.

## Methods

### Ethics statement

The 2006 Tanzania Service Provision Assessment Survey (TSPA) was implemented by the National Bureau of Statistics. Technical assistance was provided by Macro, International, with funding from the United States Agency for International Development (USAID). The method of data collection, including obtaining informed consent, was approved by local government authorities. Secondary analysis of the de-identified dataset was approved by the Institutional Review Board of Columbia University, in protocol IRB-AAAF3452(Y3M01). The dataset is available at http://dhsprogram.com/.

### Consent statement

Data for each facility were collected from the facility in-charge and the most knowledgeable person(s) available for each service assessed in the facility audit. Verbal informed consent was obtained from the facility in-charge, from all respondents for the facility audit questionnaires, and from observed and interviewed providers and clients. Each respondent was read a statement explaining the study aims, that the facility was selected randomly, and that no patient names would be recorded or shared. Respondents were informed that their participation was voluntary, that the information they provided might be used by the Ministry of Health or other organizations in order to plan and study health services, and that the name of each facility would be removed from the dataset. Verbal consent to participate from each respondent, if provided, was noted on the questionnaire with the interviewer’s signature.

### Indicators and variables

The 2006 TSPA provides a total of over 3200 indicators of health system functioning, all of which are related to the concept of system “readiness” to deliver health care services. The TSPA assessed the capacity of health facilities to provide quality maternal and child health services as well as HIV/AIDS care [11]. A subset of 200 of these measures were selected to represent system readiness to provide services in terms of the six building blocks of health system strength identified by the World Health Organization (WHO) [12]. The building blocks are indicators of the strength, presence or functioning of (1) accessible service delivery activities, modalities, and options; (2) the availability of the health workforce and worker capability to provide care; (3) mechanisms for providing information for decision-making; (4) functioning systems for procuring, distributing, and managing essential medical products, vaccines and technologies; (5) procedures of planning, financing, and budgeting; and (6) capacities to develop and sustain systems of leadership, supervision and governance [12]. The selection of the 200 variables for this analysis was based on clinical review of TSPA variables that would be accessible as checklist items if our results were to be used for routine clinical monitoring. That is, a supervisory checklist could be designed that would match corresponding SPA indicators and also routinely available information on the service delivery readiness of health facilities. Taken together, these sets of indicators could be used to evaluate the climate of clinical readiness in rural Tanzania.

### The statistical procedure

Principal components analysis (PCA) has been used extensively to construct single scale measurements of socioeconomic status [13]. First proposed over a century ago [14], and used widely for econometric applications, PCA has had only limited application to the measurement of health system readiness [15]^1^. We employ PCA to reduce the large volume of TSPA indicators to a few indices appropriately constructed from the common variance of a specified set of health system strength indicators.

We employ PCA to transform the set of correlated variables into a reduced number of uncorrelated variables known as principal components [16]. Indicators used in the PCA were represented in both discrete and continuous formats, as recommended from studies of the construction of wealth indices that conclude PCA is suitable for use with a mix of discrete and continuous data [17]. In accordance with a simulation study comparing the use of PCA to analyze binary and ordinal variables measuring socioeconomic status [18], ordinal variables were used when possible and categorical variables were converted into a set of dummy variables when there was no information about the ordering of categories [13].

PCA is a technique that estimates a subset of variables that optimally represent the commonality of a more general set of indicators. Following the general linear model, composite indices that emerge from PCA are “orthogonal,” that is, uncorrelated with one another, but representative of the indicators that coefficients represent. For example, if all input indicators are continuous variables, the principal component is a construct that is the least square common factor for the best fitting variable that would be “explained” by the set of indicators. Individual indicators are associated with a least square coefficient that defines the relationship of the indicator to the common factor. The most common example in the literature is the use of PCA to derive estimates of economic status in settings where no single reliable variable can capture relative family wealth. Analysts compile a checklist of wealth indicators and estimate their underlying commonality with PCA [19].

With PCA, a single variable is generated that describes the commonality of a set of related indicators. In the case of health systems research, this factor is “system readiness” as indicated by the six pillars of health systems strength and various correlated indicators of system inputs. A PCA scale to be constructed from the variables, *S*, will represent the best linear representation of their common variance: the principal component. Thus, the first principal component accounts for as much of the variability in the data as possible, and each succeeding component that is estimated in turn has the highest variance possible under the constraint that it be orthogonal to (that is, uncorrelated with) the preceding components. Each indicator used in the PCA estimation generates a corresponding coefficient representing the relationship of the indicator with the principal component. Multiplying each of the coefficients by corresponding indicators associated with a particular facility and summing the products, produces a relative health system readiness index for each estimated component that can be assigned to each health facility. These estimated coefficients λ, representing the estimated contribution of each indicator to overall readiness, are presented for each indicator in Table A1. Thus, given the estimated principal coefficients for K indicators of health system readiness, each individual facility has a corresponding estimate of the readiness of system exposure that would be given by:

1 $$ {S}_{Ai0}=\kern0.5em {\uplambda}_{1A}{s}_{1 Ai} + {\uplambda}_{2A}{s}_{2 Ai} + {\uplambda}_{3A}{s}_{3 Ai}+..\kern0.5em \dots +{\uplambda}_{KA}{s}_{KAi} $$

The overall mean for the common index **S**_**A**_ for all individual facilities will define “average system readiness” as zero and with a standard deviation of 1 so that estimates of relative readiness of each facility will range between −3 and +3 for each orthogonal principal component. By converting these z-scores into percentile estimates, equation (1) can be interpreted for facility *i* as the relative percentile of system readiness that a given facility has experienced at the time of the TSPA. A test is available for determining if S_A_, represents a sufficient indicator of the common variance or if a statistically orthogonal S_B_ must be specified owing to an additional set of coefficients that capture a significantly incremental estimate of common explained variance. Deemed the “Scree test” this procedure provides an objective basis for determining the most parsimonious set of indices for describing the common variance of a set of indicators [20].

Health facilities represented in the TSPA 2006 were categorized as either (1) hospitals and health centers (HC) or (2) dispensaries. Dispensary components were assessed separately from hospitals and HC because of the large number of variables that were available for both hospitals and HC but not for dispensaries. Furthermore, the low number of HC included in the 2006 TSPA (41) meant that they had to be grouped with the 128 hospital facilities surveyed. We removed variables from either category for which there were no data, or no variance (e.g., if all values were zero). Scree tests were performed to determine the number of components to retain for each category. For each component, we then removed variables with mean value less than .05 for all variables (binary or count), or mean value greater than 0.95 for binary variables, in order to remove the most highly skewed variables. After variables with no variance or no data were excluded from the original 200 SPA measures selected to represent WHO’s six building blocks of health system strength, a total of 141 variables remained in the hospital/HC analysis. Of these, 78 also appeared in the dispensary analysis and 63 were in the hospital/HC analysis only. A total of 86 variables were retained in the dispensary analysis, 78 of which were also in the hospital/HC analysis and 8 of which appeared in the dispensary analysis only. A total of 149 unique variables were in the dispensary and/or in the hospital/HC analysis.

Variables for each analysis are listed in the tables in the Additional file 1. To facilitate interpretation of results, each indicator was assigned to one of six categories representing the medical role of each variable in primary health care by a clinician. The categories are (1) Advanced clinical (any indicator that requires an MD or MA); (2) Basic clinical (anything that could be performed by a community health worker with a secondary level education and appropriate training); (3) Preventive (vaccination capability, etc.); (4) Basic family planning; and (5) Clinical family planning (Commodities such as IUD, subdermal, or other surgical); and (6) Administrative.

## Results and discussion

**Fig. 1:**
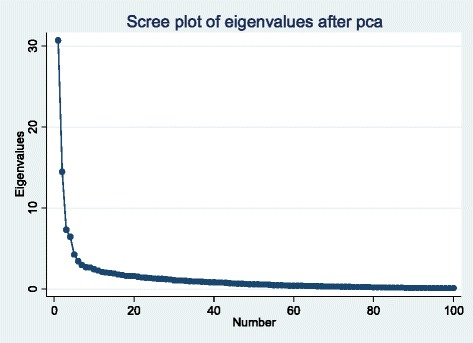
Scree plot of eigenvalues for hospitals and health centers

**Fig. 2:**
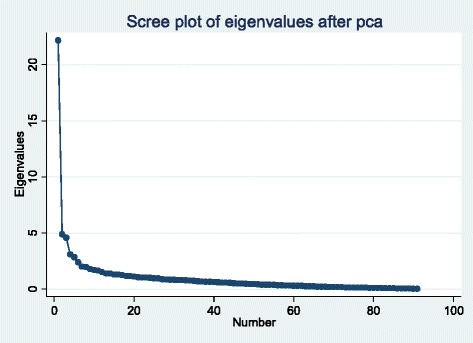
Scree plot of eigenvalues for dispensaries

From the TSPA, 169 facilities were categorized as hospitals/HCs and 442 as dispensaries. Two components extracted from 141 variables from hospitals/HCs explained 32 % of the common variance (Fig. 1), whereas one component, explaining 26 % of common variance, was retained for dispensaries (Fig. 2). Eigenvalues for each component explain how much of the common variance in the data that can be explained by a given component. Components one and two, combined, explain 32 % of the common variance among all hospitals and HC (Fig. 1), while a single component explains 26 % of the common variance for dispensaries (Fig. 2). For each component, the relative size of coefficients, whether negative or positive, portray their relative weight in explaining what all of the coefficients have in common. Clinical review of these relative weights has determined that they are indicators of contrasting characteristics of care that should be jointly taken into account when clinical readiness is described. Characteristics of each component are described below.

**Fig. 3:**
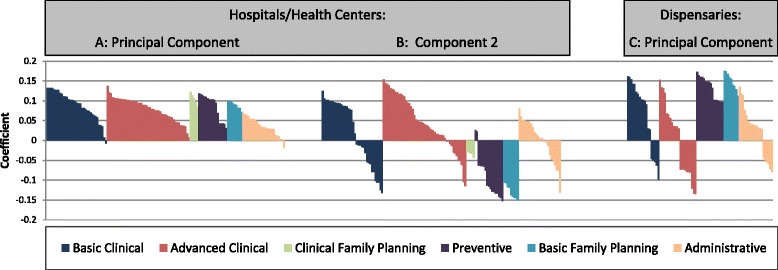
PCA loadings by category of medical role and variable rank order for hospital/health center (Part **a**, Part **b**) and dispensary analyses (Part **c**)

The principal component is weighted by preventive measures and indicators of basic primary health care capabilities that all facilities should have as essential elements of care. For hospitals/HCs, the principal component is characterized most strongly by the availability of HIV medicines, obstetric services, clinical family planning methods, vaccines and facility based vaccination services. Among basic clinical aspects of primary health care, the score is mainly determined by whether a facility has valid stores of HIV treatment drugs and basic supplies such as sterile gloves and blank partograph forms. Obstetric and newborn services such as c-sections, blood transfusions and infant resuscitation and relatively high presence of midwives are those variables scored most prominently among advanced clinical aspect of care. Availability of basic oral and injectable as well as clinical family planning services, i.e., longer acting and permanent methods, are prominent in the principal component. Facility based preventive services such as vaccination and antenatal care feature prominently in the component, while vaccination outreach is not an important part of the principal component. All variables in the principal component are listed in Additional file 1: Table S1, sorted by their medical role in primary health care and coefficient size. Figure 3 Part a, provides a visual overview of these aspects of the component.

The second component is also representative of essential health service capabilities, but is weighted more heavily by indicators of curative care functionality. In contrast to the principal component, the second component is characterized by loadings that connote a presence of higher level staff such as registered nurses, midwives, medical assistants, surgeons, obstetricians, gynecologists, pediatricians and anesthetists, as well as advanced laboratory capacity. Component 2 was not characterized by obstetric or family planning services. Among basic clinical aspects of care, component 2 is characterized by availability of HIV drugs, the newer (in 2006) combined malaria treatment Coartem and broad spectrum antibiotics. Basic supplies associated with primary health care and with obstetric and newborn care bring down the index score on component 2, as does the availability of preventive services and basic or clinical family planning services. Administrative aspects of primary health care did not feature prominently in either component. All variables in component 2 are listed in Additional file 1: Table S2, sorted by medical role in primary health care and coefficient size. Figure 3 Part b, provides a visual overview of these aspects of the component.

For dispensaries, the principal component portrays a profile of health service readiness focused on prevention and delivery that is quite different from either of the two major components describing hospitals and health centers. The dispensary principal component is characterized by very basic newborn care as well as preparedness for delivery. However, presence of higher level staff such as midwives and clinical officers or advanced laboratory services factor negatively into the component. The component is strongly associated with vaccine availability and provision both at the facility and through outreach. Availability of basic family planning services feature prominently in the component, while clinical family planning modalities could not be included in the analysis because so few of facilities offered these clinical services. Variables portraying readiness to diagnose malaria are strongly and negative associated with the component. The three variables reaching a negative loading less than or equal to −0.10 were each advanced clinical variables related to malaria testing, specifically whether the dispensary had a laboratory with a field stain glass slide malaria test, whether glass slides and covers were available in the lab, and whether the facility had rapid diagnostic tests for malaria available. Finally, the principal component prominently scores aspects of administration that portray strong ties to the community. Dispensaries that receive referrals from traditional birth attendants and have frequent routine meetings between staff and the community have a higher principal component score. Loadings for variables for the dispensary principal component are listed in Additional file 1: Table S3, sorted by medical role in primary health care and coefficient size. Figure 3 Part c, provides a visual overview of these aspects of the component.

## Conclusions

PCA of complex facility survey data generates composite scale coefficients that can be used to reduce a list of disparate indicators into composite indices for comparative analysis of clinical readiness, or for multi-level analysis of the impact of clinical capability on health outcomes or on survival. PCA cannot be applied mechanically. If a system is comprised of a service provision hierarchy, with expectations that referral care and specialized care will work their way up through the structure of the system, then PCA should be separated by level in the health system, as we have pursued in this analysis. The underlying assumption of PCA application to systems analysis is that units under observation have a common, but unmeasured, identity. If the policy and programmatic framework of a setting generates facilities that are planned and implemented as fundamentally different features of the system, then the PCA analysis should be consistent with this context.

PCA of a subset of indicators from the TSPA was performed to create three quantitative composite indices. All three components are representative of the climate of “readiness.” An evaluation of service capability would necessarily include estimation of a facility’s capability relative to these components of essential components of primary health care capacity. Thus, rather than sifting through 141 indicators for a hospital or HC, or 86 for a dispensary, relative readiness can be defined by three indices, each portraying a contrasting, but essential, component of the climate of care.

Estimated coefficients, based on a national representative sample facility survey, could be used, as expressed equation (1), to calculate relative strength of readiness scales for a given health facility, thereby facilitating routine monitoring and evaluation functions at the local, district, or regional levels. If the purpose of an investigation is to describe the climate of care relative to a national standard, PCA is the appropriate framework for estimation. However, if analyses are designed to compare facilities within a locality, statistical power problems would compromise the utility of the procedure, just as sample size constraints limit the utility of any application of the general linear model.

Given the estimation of the model 1 coefficients, any clinic can be scored relative to the national standard that our paper has derived. For the national TSPA, S_Ai0_, S_Bi0_ and S_Ci0_ each have a mean of zero and a standard deviation of 1. However, any clinic in Tanzania can be scored for “relative service readiness” by (1) compiling a checklist with the 141 indicators of our model for hospitals or HC, or the 86 indicators for dispensaries, (2) multiplying values of observed indicators for a given clinic by our corresponding estimated coefficients and (3) summing products to yield scores for the clinic estimation of S_Ai0_ and S_Bi0_ for hospitals and HC, and S_Ci0_ for dispensaries, that are either greater than zero (“stronger” than the national average) or less than zero (“weaker” than the national average for each component). If routine service monitoring compiles indicators for facilities, such as the SPA indicators used in this analysis, then the survey linkage problem is solvable: Computing composite scores for facilities in sample clusters would provide statistically tractable indicators of health systems strength that could be incorporated in multilevel statistical models of the health systems determinants of health status and survival. Such analyses would enhance the implementation research utility of both SPA facility data and DHS household survey data. Moreover, checklists compiled over time and used to generate a time series in *S*_*Ai*0_, *S*_*Bi*0_, *S*_*Ci*0_, provide an objective indication of whether a given facility has systems readiness capabilities that are improving or deteriorating with passing time, relative to other facilities where the checklist has been administered.

## Additional file

Additional file 1: Table S1. PCA loadings for Hospitals/Health Centers Principal Component , sorted by medical role in primary health care and size of coefficient. **Table S2.** PCA loadings for Hospitals/Health Centers Component 2, sorted by medical role in primary health care and size of coefficient. **Table S3.** PCA loadings for Dispensaries Principal Component, sorted by medical role in primary health care and size of coefficient. (DOCX 38 kb)

## Abbreviations

DHS: Demographic and Health Survey
HC: Health Center
MDG: Millennium Development Goal
MOHSW: Ministry of Health and Social Welfare
PCA: Principal Components Analytical
SPA: Service Provision Assessment
TSPA: Tanzania Service Provision Assessment
WHO: World Health Organization

## Footnotes

1 PCA has been used to measure levels of care in health networks and systems, although these analyses were not the focus of their published work [21].
