# Supplementary material for: Estimation of indices of health service readiness with a principal component analysis of the Tanzania Service Provision Assessment Survey
Source: BMC Health Serv Res. 2015 Dec 3;15:536. doi: 10.1186/s12913-015-1203-7 (PMC4669619; doi:10.1186/s12913-015-1203-7)
Supplement: Additional file 1: Table S1. — PCA loadings for Hospitals/Health Centers Principal Component , sorted by medical role in primary health care and size of coefficient. Table S2. PCA loadings for Hospitals/Health Centers Component 2, sorted by medical role in primary health care and size of coefficient. Table S3. PCA loadings for Dispensaries Principal Component, sorted by medical role in primary health care and size of coefficient. (DOCX 38 kb) [file 12913_2015_1203_MOESM1_ESM.docx]

**Additional file 1: Table S1 PCA loadings for Hospitals/Health Centers Principal Component , sorted by medical role in primary health care and size of coefficient**

| **Variable** |  |
| --- | --- |
| **Basic Clinical** |  |
| Facility has all valid Nevirapine syrup | 0.133 |
| Facility pharmacy has all valid Stavudine 40 | 0.133 |
| Facility has all valid Lamivudine/Stavudine 30/Nevirapine Combo | 0.133 |
| Facility has all valid Stavudine 30 | 0.132 |
| Facility has all valid Lamivudine 3TC | 0.131 |
| Facility has all valid Efavirenz 600 | 0.128 |
| Facility has all valid Lamivudine/Stavudine 40/Nevirapine Combo | 0.127 |
| Facility has all valid Nevirapine | 0.127 |
| Facility has all valid Lamivudine 3TC Syrup | 0.120 |
| Facility has all valid Zidovudine syrup | 0.117 |
| Number of days per month routine Vitamin A supplementation provided at facility | 0.112 |
| Cotton wool or gauze pad observed (for IUD or implant) | 0.111 |
| Sterile gloves observed (for IUD or implant) | 0.110 |
| Register/stock cards for contraceptives observed | 0.104 |
| Facility routinely weighs newborns | 0.102 |
| Antiseptic solution observed (for IUD or implant) | 0.102 |
| Sterile syringes and needles observed | 0.101 |
| Infant scale for newborn care functioning | 0.099 |
| Facility has all valid Zidovudine | 0.095 |
| Blood pressure apparatus for ANC/PNC observed | 0.094 |
| Blank partographs observed | 0.093 |
| Facility has all valid Chloramphenicol Oral | 0.082 |
| Trained counselors for pre and post testing for HIV present on day of interview | 0.082 |
| Facility has at least one valid Flagyl | 0.080 |
| Stethoscope observed | 0.076 |
| Facility has all valid Efavirenz 200 | 0.074 |
| Facility has at least one valid Cotrimoxazole | 0.070 |
| Facility has all valid ORS | 0.067 |
| Days per month growth monitoring is provided at facility | 0.063 |
| Suction bulb for mucus extraction functioning | 0.061 |
| Facility has all valid Quinine Oral | 0.059 |
| Thermometer | 0.039 |
| Infant scale for PNC functioning | 0.038 |
| Antibiotic eye drops or ointment chloramphenicol | 0.035 |
| Facility has all valid Coartem | 0.008 |
| Days per week STI services are available | **-0.008** |
| **Advanced Clinical** |  |
| C Sections ever performed at facility | 0.137 |
| Injectable oxytocin/syntocin available | 0.121 |
| Blood transfusion performed for maternity care within past 3 months | 0.119 |
| Bag and masks for infant resuscitation available | 0.108 |
| Injectable magnesium sulfate available | 0.108 |
| Bag or tube and mask (infant size) for resuscitation functional | 0.107 |
| Facility provides 24 hour coverage for delivery services | 0.106 |
| Table or bed for delivery available | 0.105 |
| Average number of Assistant Medical Officers at facility full time over past 3 months | 0.105 |
| Average number of Clinical Officers (other than CO Anesthetists) at facility full time over past 3 months | 0.104 |
| Parenteral anti-convulsants for pregnancy-induced hypertension administered at facility within past 3 months | 0.104 |
| Injectable ergometrine/ methergine available in delivery area | 0.103 |
| Average number of Public Health Nurses at facility full time over past 3 months | 0.103 |
| Average number of Midwives at facility full time over past 3 months | 0.101 |
| Parenteral oxytocin drugs administered at facility within past 3 months | 0.101 |
| Total number of c-sections in last 12 months | 0.099 |
| Intravenous solutions (Ringers lactate, D5NS, or NS) available in delivery service area | 0.098 |
| Blood test for anemia is routine part of ANC | 0.098 |
| Facility has lab with functioning incubator for culturing | 0.095 |
| Blood bank at facility | 0.095 |
| Ventouse (vacuum extractor) available | 0.094 |
| Urine test for protein is routine part of ANC | 0.090 |
| Manual vacuum aspiration or D & C used to remove products of conception in past 3 months | 0.090 |
| Assisted delivery conducted in this facility in past 3 months | 0.088 |
| Manual removal of placenta provided in the last 3 months | 0.085 |
| Glass slides and covers available in lab | 0.083 |
| Injectable diazepam available in delivery service area | 0.080 |
| Facility has at least one valid Methyldopa | 0.079 |
| Average number of Medical Attendants/Auxiliary Nurses at facility full time over past 3 months | 0.076 |
| Acceptable light source for pelvic exam available | 0.076 |
| Facility has rapid diagnostic tests for malaria available | 0.076 |
| Private car or bus most common transport used by women coming from home to facility for obstetric emergency | 0.074 |
| Injectable amoxicillin or ampicillin available in delivery area | 0.067 |
| Facility has all valid Chloramphenical injections | 0.066 |
| Urine test for glucose is routine for ANC | 0.066 |
| Average number of Registered Nurses/Nursing Officers at facility full time over past 3 months | 0.064 |
| Facility routinely suctions the newborn by means of catheter | 0.061 |
| Average number of Clinical Officer Anesthetists at facility full time over past 3 months | 0.059 |
| Average number of Pediatricians at facility full time over past 3 months | 0.058 |
| Average number of OBGYNs at facility full time over past 3 months | 0.053 |
| Number of days per week routinely open for outpatient curative services | 0.047 |
| Average number of Anesthetists at facility full time over past 3 months | 0.046 |
| Facility has laboratory with Giemsa glass slide malaria test | 0.046 |
| Injectable gentamycin available in delivery service area | 0.038 |
| Average number of Medical Officers at facility full time over past 3 months | 0.038 |
| Average number of Other specialists at facility full time over past 3 months | 0.037 |
| Average number of Surgeons at facility full time over past 3 months | 0.035 |
| Number of days per month sick child consultation provided at facility | 0.017 |
| <=60 minutes to nearest emergency obstetric care referral facility during the dry season | 0.008 |
| **Clinical Family Planning** |  |
| Intrauterine device provided here | 0.123 |
| Male or female sterilization services are provided here | 0.114 |
| Impants are provided here | 0.106 |
| Intrauterine devices (IUD) are all valid | 0.096 |
| Implants are all valid | 0.085 |
| **Preventive** |  |
| Number of days per month BCG vaccine provided at facility | 0.118 |
| Facility stores vaccines | 0.116 |
| Oral Polio vaccines all valid | 0.116 |
| Tetanus toxoid vaccines all valid | 0.112 |
| Number days per week ANC services available | 0.110 |
| BCG and dilutant all valid | 0.106 |
| DPT+HepB vaccine all valid | 0.104 |
| Register of vaccines observed | 0.104 |
| Vitamin A All Valid | 0.103 |
| Number of days per month DPT-HB and Polio vaccine provided at facility | 0.102 |
| Measles and diluatnt All Valid | 0.094 |
| Number of days per month Measles vaccine provided at facility | 0.070 |
| Number of days per month DPT-HB and Polio vaccine provided during outreach | 0.044 |
| Number of days per month Vitamin A supplementation provided during outreach | 0.043 |
| Number of days per month BCG vaccine provided during outreach | 0.043 |
| Number of days per month Measles vaccine provided during outreach | 0.042 |
| Number of staff received training on IMCI guidelines | 0.032 |
| **Basic Family Planning** |  |
| Combined pill provided here | 0.100 |
| Progestin-only injectable (2 or 3 monthly) provided here | 0.099 |
| Progestin pill provided here | 0.098 |
| Combined oral pill all valid | 0.093 |
| Days/week family planning services are provided at facility | 0.092 |
| Progestin-only pill all valid | 0.090 |
| Progestin-only injection (2 or 3 monthly) all valid | 0.082 |
| Male condom provided here | 0.082 |
| Male condoms all valid | 0.071 |
| **Administrative** |  |
| Facility has a functional generator and fuel on day of interview | 0.068 |
| HMIS reports on HIV deaths are observed and submitted to district | 0.066 |
| Facility has a functional ambulance that can be used for any emergency | 0.065 |
| Facility has a facility-wide review of mortality | 0.061 |
| HMIS reports on HIV deaths are observed and submitted to region | 0.055 |
| Facility has a functional ambulance with fuel | 0.054 |
| Record of management meetings observed | 0.054 |
| Management meetings occur at least once every 6 months | 0.053 |
| HMIS reports on HIV deaths are observed and submitted to national level | 0.049 |
| Evidence of quality assurance committee or staff reports is observed | 0.041 |
| HMIS reports on HIV deaths are observed and submitted to donor | 0.040 |
| Supervisor observed services within the past 6 months | 0.034 |
| Facility has a working phone or radio at all times patients are served | 0.034 |
| Facility radios or phones another facility to request transportation in obstetric emergencies | 0.033 |
| Ambulance is most common transport used by women coming from home to facility for obstetric emergency | 0.030 |
| Facility has routine meetings to review management and administrative issues | 0.030 |
| TBAs refer women to facility | 0.030 |
| Incubator functioning for newborn care | 0.030 |
| Most commonly used water source at time of interview is a protected water source | 0.029 |
| Supervisor checked registers within the past 6 months | 0.014 |
| Facility hires local vehicle to provide emergency transport | 0.013 |
| % Interviews where HMIS Guidelines Seen or Reported | 0.009 |
| Meetings with staff and community occur at least once every 6 months | 0.004 |
| Routine meetings include facility staff and community | 0.002 |
| Nearby phone available at all times | **-0.018** |

**Additional file 1: Table S2 PCA loadings for Hospitals/Health Centers Component 2, sorted by medical role in primary health care and size of coefficient**

| **Variable** |  |
| --- | --- |
| **Basic Clinical** |  |
| Facility has all valid Efavirenz 200 | 0.125 |
| Facility has all valid Coartem | 0.106 |
| Facility has all valid Efavirenz 600 | 0.102 |
| Facility has all valid Lamivudine 3TC | 0.102 |
| Facility has at least one valid Cotrimoxazole | 0.100 |
| Facility has all valid Lamivudine/Stavudine 40/Nevirapine Combo | 0.098 |
| Facility pharmacy has all valid Stavudine 40 | 0.098 |
| Facility has all valid Zidovudine syrup | 0.097 |
| Facility has all valid Zidovudine | 0.095 |
| Facility has all valid Lamivudine/Stavudine 30/Nevirapine Combo | 0.094 |
| Facility has all valid Nevirapine | 0.094 |
| Facility has all valid Lamivudine 3TC Syrup | 0.091 |
| Facility has all valid Stavudine 30 | 0.087 |
| Facility has at least one valid Flagyl | 0.087 |
| Facility has all valid Nevirapine syrup | 0.086 |
| Facility has all valid Quinine Oral | 0.081 |
| Facility has all valid Chloramphenicol Oral | 0.078 |
| Trained counselors for pre and post testing for HIV present on day of interview | 0.077 |
| Facility has all valid ORS | 0.047 |
| Suction bulb for mucus extraction functioning | 0.017 |
| Antiseptic solution observed (for IUD or implant) | **-0.010** |
| Blank partographs observed | **-0.013** |
| Cotton wool or gauze pad observed (for IUD or implant) | **-0.013** |
| Sterile gloves observed (for IUD or implant) | **-0.016** |
| Thermometer | **-0.017** |
| Sterile syringes and needles observed | **-0.032** |
| Antibiotic eye drops or ointment chloramphenicol | **-0.054** |
| Number of days per month routine Vitamin A supplementation provided at facility | **-0.057** |
| Days per week STI services are available | **-0.060** |
| Infant scale for newborn care functioning | **-0.079** |
| Infant scale for PNC functioning | **-0.080** |
| Stethoscope observed | **-0.103** |
| Blood pressure apparatus for ANC/PNC observed | **-0.106** |
| Facility routinely weighs newborns | **-0.107** |
| Register/stock cards for contraceptives observed | **-0.126** |
| Days per month growth monitoring is provided at facility | **-0.132** |
| **Advanced Clinical** |  |
| Average number of Registered Nurses/Nursing Officers at facility full time over past 3 months | 0.154 |
| Facility has lab with functioning incubator for culturing | 0.145 |
| Average number of Midwives at facility full time over past 3 months | 0.143 |
| Average number of Medical Attendants/Auxiliary Nurses at facility full time over past 3 months | 0.139 |
| Average number of Surgeons at facility full time over past 3 months | 0.132 |
| Glass slides and covers available in lab | 0.131 |
| Average number of OBGYNs at facility full time over past 3 months | 0.127 |
| Average number of Pediatricians at facility full time over past 3 months | 0.122 |
| Facility has rapid diagnostic tests for malaria available | 0.121 |
| Average number of Clinical Officer Anesthetists at facility full time over past 3 months | 0.118 |
| Average number of Anesthetists at facility full time over past 3 months | 0.117 |
| Number of days per week routinely open for outpatient curative services | 0.116 |
| Average number of Other specialists at facility full time over past 3 months | 0.111 |
| Average number of Medical Officers at facility full time over past 3 months | 0.099 |
| Facility has laboratory with Giemsa glass slide malaria test | 0.096 |
| Average number of Assistant Medical Officers at facility full time over past 3 months | 0.093 |
| Facility has at least one valid Methyldopa | 0.086 |
| Blood bank at facility | 0.080 |
| Assisted delivery conducted in this facility in past 3 months | 0.065 |
| Parenteral anti-convulsants for pregnancy-induced hypertension administered at facility within past 3 months | 0.053 |
| C Sections ever performed at facility | 0.048 |
| Average number of Public Health Nurses at facility full time over past 3 months | 0.047 |
| Injectable magnesium sulfate available | 0.047 |
| Parenteral oxytocin drugs administered at facility within past 3 months | 0.044 |
| Injectable amoxicillin or ampicillin available in delivery area | 0.042 |
| Total number of c-sections in last 12 months | 0.039 |
| Injectable oxytocin/syntocin available | 0.036 |
| Ventouse (vacuum extractor) available | 0.033 |
| Manual removal of placenta provided in the last 3 months | 0.027 |
| Average number of Clinical Officers (other than CO Anesthetists) at facility full time over past 3 months | 0.025 |
| Urine test for glucose is routine for ANC | 0.021 |
| Bag and masks for infant resuscitation available | 0.016 |
| Acceptable light source for pelvic exam available | 0.016 |
| Blood transfusion performed for maternity care within past 3 months | 0.014 |
| Manual vacuum aspiration or D & C used to remove products of conception in past 3 months | 0.014 |
| Bag or tube and mask (infant size) for resuscitation functional | 0.013 |
| Facility routinely suctions the newborn by means of catheter | 0.007 |
| Facility has all valid Chloramphenical injections | **-0.003** |
| Injectable gentamycin available in delivery service area | **-0.003** |
| Urine test for protein is routine part of ANC | **-0.009** |
| Private car or bus most common transport used by women coming from home to facility for obstetric emergency | **-0.014** |
| Injectable diazepam available in delivery service area | **-0.031** |
| Intravenous solutions (Ringers lactate, D5NS, or NS) available in delivery service area | **-0.033** |
| Blood test for anemia is routine part of ANC | **-0.039** |
| Number of days per month sick child consultation provided at facility | **-0.050** |
| <=60 minutes to nearest emergency obstetric care referral facility during the dry season | **-0.052** |
| Injectable ergometrine/ methergine available in delivery area | **-0.061** |
| Table or bed for delivery available | **-0.105** |
| Facility provides 24 hour coverage for delivery services | **-0.116** |
| **Clinical Family Planning** |  |
| Intrauterine devices (IUD) are all valid | **-0.025** |
| Implants are all valid | **-0.031** |
| Intrauterine device provided here | **-0.031** |
| Male or female sterilization services are provided here | **-0.034** |
| Impants are provided here | **-0.045** |
| **Preventive** |  |
| Number of days per month BCG vaccine provided at facility | 0.027 |
| Number of staff received training on IMCI guidelines | 0.023 |
| Number of days per month Measles vaccine provided during outreach | **-0.063** |
| Number of days per month BCG vaccine provided during outreach | **-0.064** |
| Number of days per month DPT-HB and Polio vaccine provided during outreach | **-0.065** |
| Number of days per month Vitamin A supplementation provided during outreach | **-0.066** |
| Number of days per month Measles vaccine provided at facility | **-0.076** |
| Number days per week ANC services available | **-0.113** |
| Number of days per month DPT-HB and Polio vaccine provided at facility | **-0.116** |
| Oral Polio vaccines all valid | **-0.123** |
| BCG and dilutant all valid | **-0.129** |
| Register of vaccines observed | **-0.130** |
| DPT+HepB vaccine all valid | **-0.133** |
| Vitamin A All Valid | **-0.134** |
| Facility stores vaccines | **-0.141** |
| Tetanus toxoid vaccines all valid | **-0.145** |
| Measles and diluatnt All Valid | **-0.152** |
| **Basic Family Planning** |  |
| Progestin-only pill all valid | -0.106 |
| Male condoms all valid | -0.108 |
| Progestin pill provided here | -0.118 |
| Progestin-only injection (2 or 3 monthly) all valid | -0.118 |
| Progestin-only injectable (2 or 3 monthly) provided here | -0.137 |
| Male condom provided here | -0.141 |
| Combined oral pill all valid | -0.144 |
| Combined pill provided here | -0.146 |
| Days/week family planning services are provided at facility | -0.150 |
| **Administrative** |  |
| HMIS reports on HIV deaths are observed and submitted to donor | 0.080 |
| Facility hires local vehicle to provide emergency transport | 0.060 |
| Incubator functioning for newborn care | 0.051 |
| HMIS reports on HIV deaths are observed and submitted to national level | 0.050 |
| Facility has a functional generator and fuel on day of interview | 0.048 |
| Facility has a functional ambulance that can be used for any emergency | 0.047 |
| Most commonly used water source at time of interview is a protected water source | 0.047 |
| Facility has a working phone or radio at all times patients are served | 0.042 |
| Ambulance is most common transport used by women coming from home to facility for obstetric emergency | 0.034 |
| Facility has a functional ambulance with fuel | 0.020 |
| Management meetings occur at least once every 6 months | 0.016 |
| Evidence of quality assurance committee or staff reports is observed | 0.012 |
| HMIS reports on HIV deaths are observed and submitted to region | 0.007 |
| HMIS reports on HIV deaths are observed and submitted to district | 0.006 |
| Nearby phone available at all times | 0.004 |
| Facility has a facility-wide review of mortality | 0.000 |
| Record of management meetings observed | **-0.006** |
| Facility has routine meetings to review management and administrative issues | **-0.014** |
| Supervisor observed services within the past 6 months | **-0.037** |
| % Interviews where HMIS Guidelines Seen or Reported | **-0.048** |
| Facility radios or phones another facility to request transportation in obstetric emergencies | **-0.050** |
| Supervisor checked registers within the past 6 months | **-0.063** |
| Meetings with staff and community occur at least once every 6 months | **-0.075** |
| Routine meetings include facility staff and community | **-0.075** |
| TBAs refer women to facility | **-0.131** |

**Additional file 1: Table S3 PCA loadings for Dispensaries Principal Component, sorted by medical role in primary health care and size of coefficient**

| **Variable** |  |
| --- | --- |
| **Basic Clinical** |  |
| Stethoscope observed | 0.162 |
| Register/stock cards for contraceptives observed | 0.160 |
| Blood pressure apparatus for ANC/PNC observed | 0.154 |
| Facility routinely weighs newborns | 0.143 |
| Thermometer | 0.143 |
| Infant scale for postpartum care functioning | 0.123 |
| Infant scale for newborn care functioning | 0.118 |
| Blank partographs observed | 0.111 |
| Days per month growth monitoring is provided at facility | 0.104 |
| Number of days per month routine Vitamin A supplementation provided at facility | 0.102 |
| Antibiotic eye drops or ointment chloramphenicol | 0.100 |
| Register/stock cards for medicines observed | 0.092 |
| Suction bulb for mucus extraction functioning | 0.030 |
| Facility has all valid ORS | 0.028 |
| Facility has all valid Coartem | **-0.046** |
| Days per week STI services are available | **-0.050** |
| Facility has at least one valid Flagyl | **-0.055** |
| Facility has all valid Quinine Oral | **-0.064** |
| Facility has all valid Chloramphenicol Oral | **-0.099** |
| **Advanced Clinical** |  |
| Table or bed for delivery available | 0.153 |
| Facility provides 24 hour coverage for delivery services | 0.134 |
| Injectable ergometrine/ methergine available in delivery area | 0.131 |
| Injectable diazepam available in delivery service area | 0.120 |
| Facility has all valid Chloramphenical injections | 0.069 |
| Intravenous solutions (Ringers lactate, D5NS, or NS) available in delivery service area | 0.066 |
| <=60 minutes to nearest emergency obstetric care referral facility during the dry season | 0.057 |
| Facility routinely suctions the newborn by means of catheter | 0.045 |
| <=60 minutes to nearest emergency obstetric care referral facility during the wet season | 0.037 |
| Bag and masks for infant resuscitation available | 0.035 |
| Bag or tube and mask (infant size) for resuscitation functional | 0.035 |
| Manual removal of placenta provided in the last 3 months | 0.030 |
| Average number of Registered Nurses/Nursing Officers at facility full time over past 3 months | **-0.073** |
| Average number of Public Health Nurses at facility full time over past 3 months | **-0.073** |
| Average number of Clinical Officers (other than CO Anesthetists) at facility full time over past 3 months | **-0.073** |
| Facility has laboratory with Giemsa glass slide malaria test | **-0.076** |
| Number of days per week routinely open for outpatient curative services | **-0.080** |
| Facility has at least one valid Methyldopa | **-0.080** |
| Average number of Assistant Medical Officers at facility full time over past 3 months | **-0.081** |
| Facility has laboratory with Field Stain glass slide malaria test | **-0.122** |
| Glass slides and covers available in lab | **-0.134** |
| Facility has rapid diagnostic tests for malaria available | **-0.136** |
| **Preventive** |  |
| Facility stores vaccines | 0.173 |
| Measles and diluatnt All Valid | 0.164 |
| Vitamin A All Valid | 0.161 |
| Tetanus toxoid vaccines all valid | 0.160 |
| Register of vaccines observed | 0.157 |
| DPT+HepB vaccine all valid | 0.150 |
| Oral Polio vaccines all valid | 0.149 |
| BCG and dilutant all valid | 0.148 |
| Number days per week ANC services available | 0.144 |
| Number of days per month DPT-HB and Polio vaccine provided at facility | 0.132 |
| Number of days per month Vitamin A supplementation provided during outreach | 0.102 |
| Number of days per month DPT-HB and Polio vaccine provided during outreach | 0.102 |
| Number of days per month BCG vaccine provided at facility | 0.101 |
| Number of days per month BCG vaccine provided during outreach | 0.098 |
| Number of days per month Measles vaccine provided during outreach | 0.097 |
| Number of days per month Measles vaccine provided at facility | 0.097 |
| **Basic Family Planning** |  |
| Progestin-only injectable (2 or 3 monthly) provided here | 0.176 |
| Combined pill provided here | 0.175 |
| Days/week family planning services are provided at facility | 0.168 |
| Male condom provided here | 0.160 |
| Combined oral pill all valid | 0.155 |
| Progestin-only injection (2 or 3 monthly) all valid | 0.140 |
| Progestin pill provided here | 0.138 |
| Male condoms all valid | 0.129 |
| Progestin-only pill all valid | 0.112 |
| **Administrative** |  |
| TBAs refer women to facility | 0.135 |
| Routine meetings include facility staff and community | 0.119 |
| Meetings with staff and community occur at least once every 6 months | 0.113 |
| Record of meetings with staff and community observed | 0.077 |
| % Interviews where HMIS Guidelines seen or reported | 0.063 |
| Facility radios or phones another facility to request transportation in obstetric emergencies | 0.047 |
| Supervisory checklist of health system components observed for quality assurance | 0.043 |
| Supervisor checked registers within the past 6 months | 0.040 |
| % Interviews where any special HMIS training seen or reported | 0.040 |
| Management meetings occur at least once every 6 months | 0.038 |
| Supervisor observed services within the past 6 months | 0.037 |
| Periodic audit of medical records or service registers observed for quality assurance | 0.032 |
| Facility has routine meetings to review management and administrative issues | 0.031 |
| Record of management meetings observed | 0.029 |
| Facility has a working phone or radio at all times patients are served | **-0.045** |
| Referral document observed | **-0.050** |
| Facility hires local vehicle to provide emergency transport | **-0.054** |
| Facility has a functional ambulance that can be used for any emergency | **-0.059** |
| Most commonly used water source at time of interview is a protected water source | **-0.072** |
| Facility has a functional generator and fuel on day of interview | **-0.079** |
